# Editorial: Single-cell tools to combat antimicrobial resistance

**DOI:** 10.3389/fmicb.2023.1256107

**Published:** 2023-07-18

**Authors:** Jiabao Xu, Li Cui, Yizhi Song

**Affiliations:** ^1^Division of Biomedical Engineering, James Watt School of Engineering, University of Glasgow, Glasgow, United Kingdom; ^2^Key Laboratory of Urban Environment and Health, Fujian Key Laboratory of Watershed Ecology, Institute of Urban Environment, Chinese Academy of Sciences, Xiamen, China; ^3^Suzhou Institute of Biomedical Engineering and Technology, Chinese Academy of Sciences, Suzhou, China; ^4^Division of Life Sciences and Medicine, School of Biomedical Engineering, University of Science and Technology of China, Suzhou, China

**Keywords:** antimicrobial resistance (AMR), single-cell, single-cell analyses, heterogeneity, Raman/IR spectroscopy

In his Nobel lecture upon receiving the Nobel Prize in Physiology or Medicine in 1945, Sir Alexander Fleming made a remarkably prescient prediction regarding the potential repercussions of the improper use of penicillin. He cautioned that such misuse would inevitably result in antibiotic resistance and treatment failures. Now, after several decades have elapsed, we find ourselves grappling with a shortage of effective options in antibiotic arsenals when confronted with numerous perilous pathogens. Projections indicate that if the current trend of antibiotic resistance prevalence continues to escalate, and if no new efficacious therapies are devised, an estimated 10 million deaths worldwide could occur due to superbug infections by 2050.

Effectively addressing this challenge necessitates a comprehensive understanding of the underlying mechanisms of resistance, as well as the development of innovative strategies to combat antimicrobial resistance (AMR). Recent advancements in single-cell tools have yielded valuable insights into the heterogeneity of microbial populations and offered a significant advantage in reducing the turnaround time (TAT) in current clinical diagnostic protocols. This is achieved by circumventing the need for protracted cultivation processes, which can take several days or even weeks to yield definitive results. Single-cell methodologies expedite the diagnostic process substantially, allowing for prompt intervention and targeted treatment.

Among these methodologies, single-cell spectroscopic techniques, specifically Raman spectroscopy and optical photothermal infrared (O-PTIR) spectroscopy, have garnered particular attention in this Research Topic. Raman and IR spectroscopic techniques can generate highly specific vibrational fingerprints of biomolecules, providing a snapshot of the presence and phenotypic identity of pathogens. By joining vibrational spectroscopy with advanced computational methods, researchers can unravel the intricate relationship between molecular signatures, thereby establishing the foundation for the rapid identification of pathogens at the single-cell level. Xu et al. demonstrated the rapid (five cells per sample; acquisition time 2 s per cell) and accurate (100%) classification of 94 clinical isolates resulting from fungal infections, reporting a sample-to-result time within 1 h. Lu et al. reported signature changes in the Raman spectra of 12 species of pathogenic bacteria, as well as in antibiotic-sensitive and resistant strains of *Acinetobacter baumannii*, with a higher nucleic acid/protein ratio observed in the sensitive strains.

Beyond pathogen identification, an elegant approach to measuring microbial metabolism under the influence of antibiotics via Raman spectroscopy involves culturing cells in partially deuterated water. As metabolically active cells incorporate deuterated water molecules into new biosynthetic products, they create new carbon–deuterium (C–D) bonds. This technique, known as Raman-deuterium isotope profiling (Raman-DIP), has proven to be remarkably swift, with fast-growing cells requiring <20 min in deuterated water to exhibit active metabolism. In their study, Wang et al. further refined the current Raman-DIP approach by incorporating a high concentration of sodium acetate as an inhibitor of pathogen replication, while simultaneously preserving their metabolic activity. This breakthrough allowed for the rapid quantification of viable bacteria in urinary tract infections, within a timeframe of <3 h.

In contrast to single-cell Raman modalities, research utilizing IR spectroscopy has predominantly been conducted at a population level, primarily due to limitations imposed by the diffraction limit when employing longer wavelengths. Recent advances in O-PTIR spectroscopy have circumvented this constraint by probing the photothermal effects of IR radiation. Shams et al. were the first to incorporate DIP into O-PTIR spectroscopy, thereby showcasing the characteristic C-D stretching at 2,159 cm^−1^ and detecting AMR at the single-cell level.

In addition to spectroscopic techniques, the collection of articles in this Research Topic contributes to an enhanced understanding of the mechanisms of AMR at the single-cell level. Nordholt et al. synthesized fluorescent analogs to the disinfectant active agent, quaternary ammonium compounds (QACs), and elucidated their mode of action through flow cytometry and fluorescent microscopy.

The articles featured in this Research Topic underscore the power and potential of single-cell tools in combating AMR. Many of these exemplify the capability of single-cell techniques to revolutionize clinical microbiology by rapidly and accurately identifying pathogens' type and quantity, and obtaining the antimicrobial susceptibility profile, ultimately resulting in reduced TAT and improved patient outcomes. The showcased advancements in spectroscopic tools, machine learning, and microscopy techniques offer researchers and clinicians powerful tools to study and combat AMR at the single-cell level. We hope that these contributions will inspire further research and innovation in this field, ultimately leading to the development of novel interventions to address the growing threat of AMR.

## Author contributions

All authors listed have made a substantial, direct, and intellectual contribution to the work and approved it for publication.

